# Incidence and clinicopathologic features of H3 K27M mutations in adults with radiographically-determined midline gliomas

**DOI:** 10.1007/s11060-019-03134-x

**Published:** 2019-03-12

**Authors:** Karisa C. Schreck, Surabhi Ranjan, Nebojša Skorupan, Chetan Bettegowda, Charles G. Eberhart, Heather M. Ames, Matthias Holdhoff

**Affiliations:** 1 Sidney Kimmel Comprehensive Cancer Center at Johns Hopkins, 201 North Broadway, Viragh Building, 9th floor, Post Box 3, Baltimore, MD 21287 USA; 2 Department of Neurology, Johns Hopkins University School of Medicine, Baltimore, MD USA; 3 Department of Oncology, Johns Hopkins University School of Medicine, Baltimore, MD USA; 4 Department of Neurosurgery, Johns Hopkins University School of Medicine, Baltimore, MD USA; 5 Department of Pathology, Johns Hopkins University School of Medicine, Baltimore, MD USA; 6 Department of Pathology, University of Maryland School of Medicine, Baltimore, MD USA

**Keywords:** Diffuse midline glioma, Glioma, H3 K27M, IDH1

## Abstract

**Purpose:**

H3 K27 mutations, most commonly in H3F3A, are common in diffuse midline glioma. The exact frequency of these mutations in adults with gliomas in the midline location is unknown. This study was conducted to define the incidence of H3 K27M mutations in this location and to compare clinicopathological features with those of patients who do not harbor this mutation.

**Methods:**

Consecutive glioma cases from 2007 to 2017 were screened for gliomas in the midline location. Immunohistochemistry was performed on all available tissue for mutations of H3 K27M, IDH1, and ARTX.

**Results:**

Of 850 gliomas screened, 163 cases had midline glioma on MRI. Sufficient FFPE tissue was available for 123 cases (75%). H3 K27M mutation was identified in 18 of 123 cases (15%). All except one H3 K27M-mutant tumors were WHO grade III or IV on histology, while non-mutant tumors encompassed all four grades. The most common midline locations for H3 K27M-mutated tumors were midbrain (2/3; 67%), pons (4/11; 36%), and cerebellum (6/24; 25%). As compared to H3 K27M-wildtype tumors, there were no differences in age at diagnosis, sex, tumor grade, contrast enhancement on MRI, extent of resection, or treatment received. In this cohort, median survival was longer for patients with H3 K27M-mutated tumors (n = 18; 17.6 months) compared with high-grade wildtype tumors (n = 74; 7.7 months, p = 0.03).

**Conclusions:**

H3 K27M mutations are common in midline gliomas in adults and can present in all midline locations. Survival comparison between H3 K27M-mutant and wildtype midline gliomas suggests that survival may be similar or possibly improved if the mutation is present.

## Introduction

Diffuse midline gliomas are aggressive tumors, which were recently found to harbor a K27M mutation in *HIST1H3B*/*C* and *H3F3A* genes which code for mutant histone protein H3.1 and H3.3 respectively [1]. Diffuse midline gliomas with the H3 K27M mutation, due to their unique molecular signature and clinical features, are now recognized as a separate entity in the 2016 World Health Organization Classification of tumors of the central nervous system [2]. These tumors are found in midline locations such as the brainstem, thalamus, cerebellum and spinal cord, and are identified primarily in children. The majority of pediatric midline gliomas harbor the H3 K27M mutation, including the historical subgroup of diffuse intrinsic pontine gliomas (DIPGs). Even within pediatric DIPGs, however, the presence of H3 K27M mutation confers a worse prognosis as compared to H3 wildtype cases [3, 4]. Extrapolating from the clinicopathologic features of DIPGs and the poor prognosis seen in pediatric diffuse midline gliomas with H3 K27M mutations, the presence of an H3 K27M mutation in an infiltrating astrocytoma of the midline automatically confers a grade IV status [2, 5].

Historically, diffuse midline gliomas were predominantly recognized as pediatric brain tumors. With increased testing for the H3 K27M mutation in the context of wider sequencing efforts and integration of H3 K27M staining of tissue, these tumors are now being diagnosed across all age groups. Characterization in pediatric patients has demonstrated that this mutation is associated with a worse prognosis [3, 6]. Histologic identification of these malignancies, however, can be challenging given the difficulty of accessing tumor material for biopsy. Unique radiographic features have not been identified to aid in non-invasive diagnosis [7], but CSF circulating tumor DNA may prove a useful tool in the future [8].

Given the novelty and rarity of the H3 K27M mutation in adult brain tumors, the incidence and clinical behavior of these tumors in adults is still relatively unknown. There have been several series of H3 K27M mutated tumors in adults, which have suggested this mutation may be more prevalent in tumors of the spinal cord or thalamus in adults as compared to pediatric patients [9–12]. Two series of pathologically-midline cases showed an incidence of 50–60% H3 K27M mutations in midline gliomas in adults located in the brainstem or thalamus [11, 13].

The total proportion of H3 K27M mutation in adult midline gliomas, remains unclear. In addition, there is a lack of clarity regarding the clinical behavior of these tumors in adults and how their behavior differs compared to H3 wildtype gliomas in that location. Currently, adult cases of diffuse midline gliomas are generally treated as if they behave as aggressively as their pediatric counterparts and merit a WHO grading of IV, irrespective of the actual grade found in microscopy [10, 13], though not all series support this approach [9].

In the current study, we retrospectively identified all adult midline glioma cases at our institution (2007–2017) and immunohistochemically determined H3 K27M mutation status in all cases with remaining tissue. In addition, we compared clincopathological features of adult midline glioma patients with and without H3 K27M mutation.

## Methods

### Case identification

This study was conducted with the approval of the Johns Hopkins Institutional Review Board. Consecutive cases of adults with histologically-confirmed gliomas between 1/1/2007 and 8/31/2016 were identified using the Sidney Kimmel Comprehensive Cancer Center’s cancer registry. Brain MRI images obtained at the time of initial diagnosis or at time of first surgery were reviewed by one of two neurologists (K.S. or S.R.) to determine the location of the gliomas. Cases with a primarily midline location were identified and categorized by location: brainstem, thalamus, basal ganglia, corpus callosum, spinal cord, or cerebellum. Clinical data were collected on patient demographics, tumor histopathology, extent of surgical resection, chemotherapy and radiation treatment, time to progression, and date of death.

### Tissue preparation

Tissue from cases with sufficient quantities of stainable formalin-fixed paraffin embedded (FFPE) tissue were integrated into a tumor tissue microarray (TMA). From the remaining cases, 3–5 unstained slides were cut for immunohistochemical evaluation.

### Immunostaining

Unstained slides from the TMA and other paraffin embedded tumor blocks were cut at 7 μm in the Johns Hopkins reference core and tissue microarray core laboratories. Tissue was stained with a clinically validated polyclonal antibody against H3.3 K27M (ABE419, Millipore, Billercia, MA), IDH1 R132H and ATRX (Sigma), using a Ventana automated stainer and standard clinical protocols for Johns Hopkins Pathology [14, 15]. Immunostaining was interpreted by a neuro-pathologist and stains were scored as either positive, negative, or uninterpretable (H.A.).

### Statistics

Means and standard errors of the mean were presented for normally distributed continuous measures, and medians and ranges were presented for non-normally distributed continuous measures. Pearson’s chi squared test was used for categorical variables. Simple linear regression was used for continuous measures, and the correlation coefficient was calculated (Stata/IC 15.1). Survival analysis was performed using the Gehan–Breslow–Wilcoxon test, and graphs were created using GraphPad Prism version 7.03 (2017. La Jolla, CA: Graph Pad Software, Inc).

## Results

Eight-hundred-fifty cases of adults with histologically-confirmed gliomas were identified using the Sidney Kimmel Comprehensive Cancer Center’s cancer registry. Of these, upon radiographic review 163 were located in a midline location (19%). From this cohort, 123 patients had evaluable tissue. The demographic features and histopathologic characteristics of these patients and their tumors are found in Table 1.

**Table 1:** Demographic features, histopathologic characteristics, and prior treatments of all patients

Age (mean, range)	51 (19–86) years		
Sex (female)	54 (43.9%)		
Tumor location			
Midline cortex	2 (2%)		
Corpus callosum	29 (24%)		
Basal ganglia	6 (5%)		
Thalamus	31 (25%)		
Midbrain	3 (2%)		
Pons	11 (9%)		
Medulla	3 (2%)		
Cerebellum	24 (20%)		
Spinal cord	14 (11%)		
Contrast enhancement on MRI	96/123 (78%)		

Histopathological features			
WHO grade	3 unable to be graded		
I	2 (1.6%)		
II	27 (22%)		
III	35 (28%)		
IV	56 (45.5%)		
	Absent	Present	Unknown
MGMT promoter methylation	12	8	101
ATRX mutation	84	5	34
H3 K27M mutation	104	18	0
IDH1 mutation	119	4	0
BRAF V600E	8	0	115

Treatment			
Extent of surgery			
Biopsy	78 (64%)		
Subtotal resection	33 (27%)		
Gross total resection	10 (8%)		
Unknown	2 (2%)		
Treatment			
Observation	36 (29%)		
Radiation alone	12 (10%)		
Chemoradiation	58 (47%)		
Chemotherapy alone	2 (2%)		
Unknown	15 (12%)		
Radiation	61/108 (56%)		
Radiation dose (# pts) (Gy)			
36–45	9		
46–55	12		
56–60	25		
61–65	2		

Of note, MGMT promoter methylation status was unknown in the majority of cases. While tissue was stained for both IDH1 and ATRX, the ATRX stain was deemed uninterpretable for cases in which the internal positive controls were negative (n = 33). One case showed both ATRX protein loss and H3 K27M mutation, while *IDH1* R132H mutation was mutually exclusive with H3 K27M mutation in our dataset.

Histopathologic grade spanned all IV grades, with two patients diagnosed as grade I, 27 as grade II, 35 as grade III, and 56 as grade IV in 56 cases, with three cases unable to be graded. In accord with this, 33% of patients were observed after surgical resection (N = 36), while 67% of patients received treatment at the time of diagnosis (N = 72 of 108 cases in which treatment was known).

**Fig. 1:**
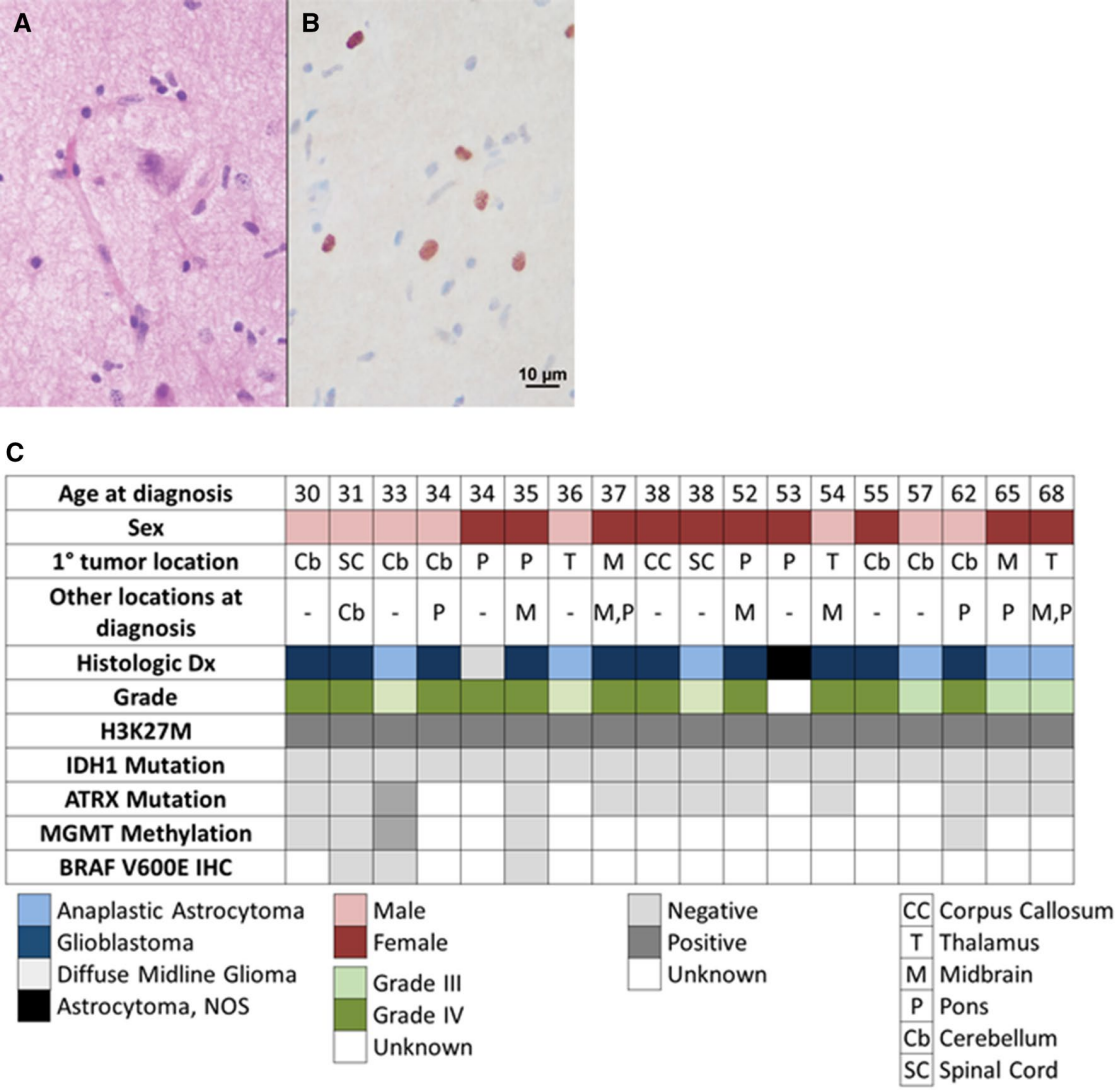
**a** Hematoxilin and eosin stain showing both normal and neoplastic cells, and **b** H3 K27M immunostain showing positive neoplastic nuclei (brown); **c** Clinical and histopathologic features of adults with H3 K27M gliomas (N = 18)

Eighteen of 123 patients had positive H3 K27M immunostaining (15%; Fig. 1). These patients were on average 45 years old at diagnosis (range 30–68 years). There were no differences between patients with H3 K27M mutated or wildtype tumors with regards to age, sex, tumor grade at diagnosis, contrast enhancement on MRI, degree of resection, or treatment received (Table 2).

**Table 2:** Comparison between demographic features and treatments received in H3 K27M-mutant tumors (N = 18) and wild type (N = 105). Values are median ± SEM

	H3 K27M WT	H3 K27M-mutant	P value
Age	53.1 ± 16.7 years	45.1 ± 12.8 years	0.51
Sex (female)	42/102 (41%)	10/18 (56%)	0.28
CE on MRI	80/96 (83%)	15/18 (83%)	0.58
Treatment			0.18
Observation	30 (29%)	6 (33%)	
Radiation alone	12 (11%)	–	
Chemoradiation	47 (45%)	11 (61%)	
Chemotherapy alone	2 (2%)	–	
Unknown	13 (12%)	1 (6%)	
RT received	61/92 (66%)	11/17 (65%)	0.77
RT dose	54.2 ± 1 Gy	54 ± 1.8 Gy	0.29
Extent of surgery			0.92
Biopsy	78 (64%)	12 (67%)	
Subtotal resection	33 (27%)	4 (22%)	
Gross total resection	10 (8%)	1 (6%)	
Unknown	2 (2%)	–	

There were notable differences in the locations of H3 K27M mutated gliomas in adults as compared with non-mutant counterparts in this study of gliomas in a radiographically midline location (p = 0.02; Table 3). Within the cases that were available for analysis, H3 K27M-mutated gliomas were most frequent in the pons (4/11, 36%), midbrain (2/3, 67%), cerebellum (6/24, 25%) and spinal cord (2/14, 14%). No mutant cases were found in basal ganglia (0/6) and in the midline cortex (0/2).

**Table 3:** Comparison between tumor location in H3 K27M-mutant tumors (N = 18) and non-mutant tumors (N = 105), with percentage of H3 K27M mutation tumors per location

Tumor location	H3 K27M WT	H3 K27M-mutant	Overall % H3 K27M-mutant
Midline cortex	2	0	0
Corpus callosum	28	1	3%
Basal ganglia	6	0	0
Thalamus	28	3	10%
Midbrain	1	2	67%
Pons	7	4	36%
Medulla	3	0	0
Cerebellum	18	6	25%
Spinal cord	12	2	14%

*WT* wild-type

**Fig. 2:**
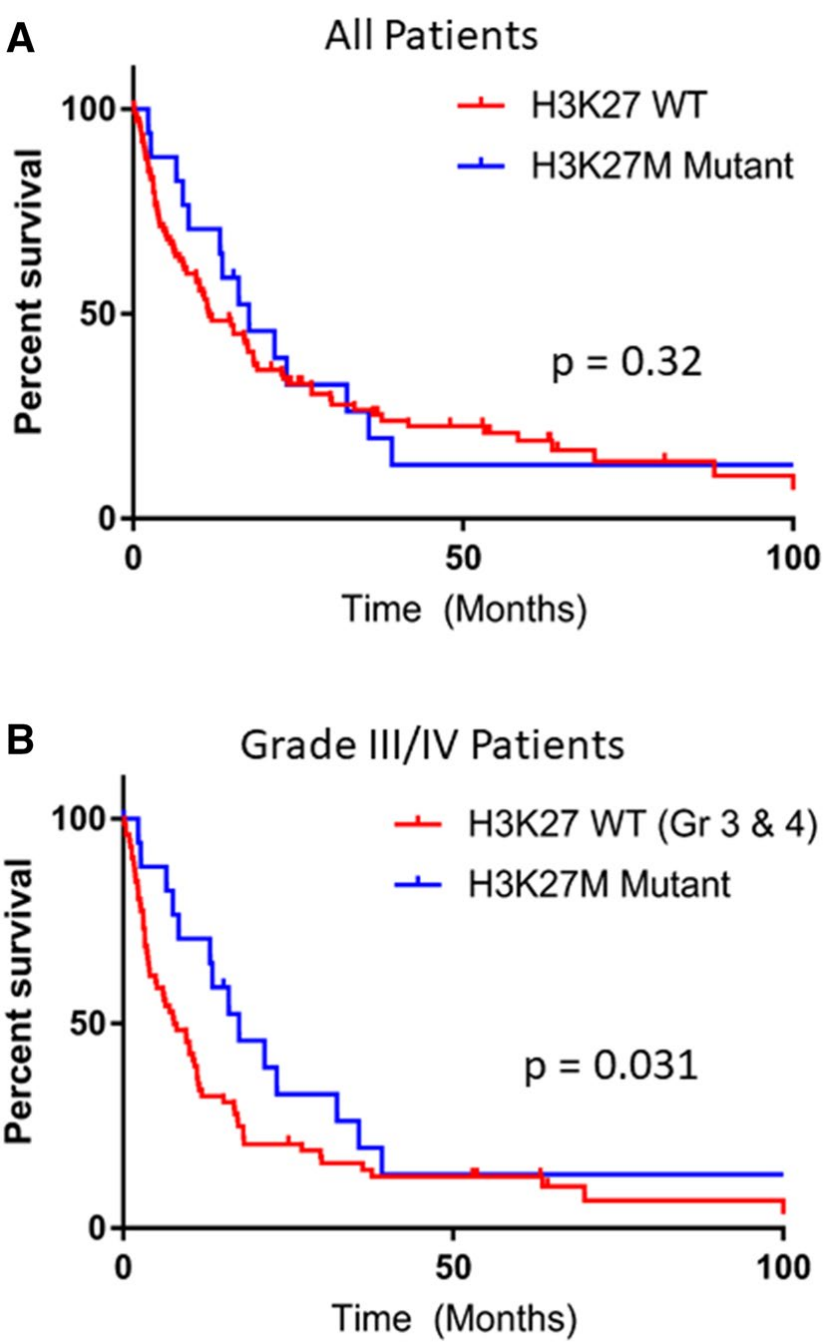
Overall survival of H3 K27M-mutated patients compared with **a** all wildtype patients (p = 0.32) or **b** Grade III or IV wildtype patients only (p = 0.031). Median survival was 17.6 months for H3 K27M-mutated, 11.3 months for all H3 K27M-non-mutant, and 7.7 months for non-mutant grade III or IV midline gliomas

Median survival for all 123 patients with radiographically-midline tumors and analyzable tissue was 13.5 months (0.1–219.1 months). Median survival for all non-H3 K27M-mutated gliomas was 11.3 months, which was not statistically different from the 17.6 month survival for H3 K27M mutated patients (p = 0.32; Fig. 2a When only high grade tumors were compared (WHO Grade III or IV), the median survival in non-H3 K27M-mutated patients was only 7.7 months [HR 1.65 (0.99–2.73)], which was significantly lower compared to that of H3 K27M-mutated patients (17.6 months) in this patient cohort and dataset (p = 0.031; HR 0.61 (0.37–1.0); Fig. 2b).

One case of a patient whose tumor stained positive for H3 K27M was particularly noteworthy, as the biopsy was concerning for but not diagnostic for astrocytoma based on histopathological analysis. The patient initially presented with imbalance on standing and was found to have an enhancing lesion in the floor of the fourth ventricle. Biopsy showed mild hypercellularity with atypical cells. The patient died 32 months later from a progressive brainstem glioma. When immunostaining was performed retrospectively as part of this study, the tissue was found to be positive for the H3 K27M mutation, which could have confirmed the diagnosis earlier for this patient.

## Discussion

Diffuse midline gliomas as defined by the WHO 2016 classification [2], occur primarily in children—though recent findings have shown that they can also occur in adults. In our study, we sought to define the incidence of H3 K27M mutations in radiographically-identified midline gliomas in adults. Our cohort is unique among previous publications: MRIs from 850 adults over a period of almost 10 years with primary gliomas were systematically reviewed and 163 cases were selected for having a glioma in one of nine pre-selected midline locations at the time of diagnosis. A relatively broad definition of midline was used, including parasagittal cortex, basal ganglia and cerebellum. Despite the broad definition, 15% of cases contained an H3 K27M mutation by immunohistochemistry. This suggests H3 K27M mutations are relatively common in adults, and far more common in this location than IDH1 mutations, which occurred in only 2% of patients in our dataset. This study highlights the importance of radiological evaluation together with histologic evaluation in the diagnosis of midline gliomas and suggests that H3 K27M immunostaining represents a higher priority stain in evaluating these neoplasms over the more common IDH mutation evaluation.

These findings also suggest that the prognosis in H3 K27M mutated diffuse midline gliomas in adults may be more nuanced than previously appreciated. H3 K27M mutations are historically associated with a dismal prognosis. In pediatric patients, gliomas with this mutation are associated with a median survival of 7–11 months [4, 10]. Due to this poor prognosis, and based on the current classification, the presence of an H3 K27M in astrocytomas automatically confers a grade IV status [2, 5]. Small series of adult patients have shown a variable prognosis, with a median survival of 8.4 months (n = 13; [10]) to 19.6 months (n = 21; [9]). In this cohort of radiographically-identified midline tumors, the H3 K27M mutation is not associated with a worse prognosis. Specifically, when compared with high-grade non-H3 K27M mutated midline gliomas from the cohort, patients with H3 K27M mutated gliomas had a better prognosis with a median survival more than double that of their comparators. These data indicate the diagnosis of an H3 K27M mutation is not universally associated with a worse prognosis as compared to H3 K27M-wt gliomas in similar locations in adults. The overall range of survivals was quite large in this group, with the longest survivor living more than 9 years after diagnosis.

This potential longevity of affected patients as well as the sensitivity of the stain may justify staining of archived tissue from patients with radiographically midline gliomas as targeted therapies against H3F3A and HIST1H3B/C are developed. Since our cohort was retrospectively identified over a period of 10 years, and the sensitivity of immunohistochemical staining of paraffin embedded tissue is known to decrease over time, we were concerned that the older samples may have more false negative stains [16]. Our older ATRX stains, for example, frequently lacked staining of internal positive control cells. Remarkably, strong specific staining for H3 K27M was present in some of the oldest specimens and the percent staining in samples after 2013 (16%, n = 43) was the same as from samples from 2013 and earlier (16%, n = 49). In aggregate, these data support the recommendation for ubiquitous staining for the H3 K27M mutation in midline gliomas, even in adult patients [10]. As H3 K27M-mutated tumors were found in some unusual midline locations, such as the corpus callosum, tumors primarily located in this region should also be tested.

Our study has several limitations and should be interpreted with caution. First, the number of H3 K27M-mutant cases was small (N = 18) and precludes definitive calculation of survival. Second, our study population is somewhat different from previous cohorts, which may account for the difference in incidence of H3 K27M mutations. Prior cohorts have identified patients with H3 K27M immunohistochemistry performed at the pathologist’s discretion for clinical purposes [9, 10]. This presents a selection bias, likely skewed toward younger patients, as the presence of the H3 K27M mutation in the elderly has only recently been appreciated. Our cohort of adults with radiographically-midline gliomas had a lower proportion of H3 K27M-mutated tumors in the thalamus and brainstem than seen in other cohorts [13]. Bias in our cohort, however, may be linked to the recruitment of cases from only a tertiary care institution—thereby favoring more acutely ill patients. Third, this analysis was limited to patients from whom tissue was present in sufficient quantities to allow for additional immunostaining for H3 K27M, IDH1, and ATRX. For example, 40 patients (25% of our radiographic cohort) did not have sufficient tissue for testing and it is unknown what proportion of those patients may have H3 K27M mutations. This may have created selection bias in our sample, as patients with a worse performance status may be less likely to have a large biopsy or resection performed. Additionally, insufficient tissue was present for sequencing confirmation of the H3 K27M mutation. While the antibody used is quite specific, we cannot rule out a false positive in the data set.

Accurate identification of H3 K27M-mutated tumors is important for accurate diagnosis and prognostication, but it may also have implications for treatment selection. Mutation-specific clinical trials are ongoing (NCT03295396, NCT02717455) or opening soon (NCT03696355). In order to maximally benefit adults with diffuse midline gliomas, increased recognition and testing of these tumors should be undertaken. Clinically, immunostaining for H3 K27M should be first line for a radiologically suspected midline glioma, particularly when limited tissue is available and/or histopathologic diagnosis is not sufficient for determining malignancy. For these purposes, this study encourages incorporation of this immunostaining in the evaluation of all midline infiltrating gliomas.
